# Impact of water deficiency on cotton ginning efficiency, fiber quality, and seed composition

**DOI:** 10.3389/fpls.2026.1761774

**Published:** 2026-02-04

**Authors:** Fang Bai, Sean P. Donohoe, Abdelraheem Abdelraheem, Nacer Bellaloui, Partson Mubvumba, Linghe Zeng

**Affiliations:** 1 The United States Department of Agriculture (USDA), Agricultural Research Service, Crop Genetics Research Unit, Stoneville, MS, United States; 2 The United States Department of Agriculture (USDA), Agricultural Research Service, Cotton Ginning Research Unit, Stoneville, MS, United States; 3 The United States Department of Agriculture (USDA), Agricultural Research Service, Environmental Soil & Water Laboratory Crop Production Systems Research Unit, Stoneville, MS, United States

**Keywords:** correlation of genotype × trait × treatment, cotton fiber quality, cottonseed protein and oil, ginning rate and ginning energy, water deficiency

## Abstract

Water deficiency is a prevalent abiotic stress that significantly constrains cotton productivity worldwide. This study aimed to evaluate the impact of water deficiency on ginning efficiency, fiber quality, and seed composition in cotton (*Gossypium* spp.). Ten cotton genotypes were assessed under irrigated and non-irrigated field conditions. Water deficiency markedly reduced plant height and the number of bolls, with genotypes MD 52ne, MD 25-26ne, and 1517–99 displaying high sensitivity, whereas CIM 432 exhibited notable water deficiency tolerance. Ginning efficiency analysis showed a general reduction in energy requirements under water deficiency, particularly in MD10-5, MD 15, and MD 52ne. CIM 432, however, maintained high boll numbers, and stable ginning rate and ginning energy under stress. Fiber quality traits such as length, strength, and uniformity were adversely affected by water deficiency across most genotypes, although CIM 432, MD 15 and 84524 showed greater stability. Correlation analyses under water deficiency revealed strong positive associations among fiber length, strength, and uniformity, along with a significant negative correlation between lint percentage and oil content, suggesting a trade-off between lint yield and seed oil accumulation. Cottonseed composition analysis indicated that when oil content declined under water deficiency, protein and seed fiber levels remained relatively unaffected. Significant genotypic variation was observed for most traits, with minimal genotype-by-treatment interactions, indicating consistent genotype performance across irrigated and non-irrigated treatments. Overall, CIM 432 emerged as a robust candidate for breeding water deficiency-tolerant cotton, combining agronomic resilience with stable fiber quality. These findings underscore the complexity of genotype-water deficiency stress interactions and highlight the importance of integrated phenotypic assessment for developing water deficiency-tolerant cotton varieties.

## Introduction

Cotton (*Gossypium* spp.) is a vital crop in global agriculture due to its valuable fiber and seed-derived products (Wang et al., 2020, 2023). While cotton is a drought-resistant crop, water deficiency can lead to changes in the plant (Saeed et al., 2011, 2024). St Aime et al. (2021) reported a positive correlation between root architectural and water use efficiency in cotton. Drought-tolerant cultivars often exhibit increased lower-root area and root length density. For instance, Guo et al. (2024A, B) documented a 34% increase in lateral root length and a 15% increase in maximum root depth under drought conditions, enabling enhanced water acquisition from deeper soil layers. Above ground, drought stress leads to reduced turgor pressure, diminished leaf area, and limited stem elongation. Pettigrew (2004) observed a 35% decrease in leaf area index (LAI) under moderate water deficiency, accompanied by reductions in biomass due to impaired carbon assimilation. Stomatal closure to prevent water loss further limits photosynthetic capacity, compounding the impact on vegetative growth and reducing the plant’s ability to support reproductive development (Abdelraheem et al., 2018).

The reproductive phase is especially susceptible to water deficiency stress. Water deficiency during flowering and boll formation frequently induces floral abortion and boll shedding, as the plant reallocates resources to survival rather than reproduction. Studies by Bauer et al. (2012) and Snowden et al. (2014) demonstrated that yield losses are most pronounced during flowering due to high water demand. Although water requirements decrease at boll maturity, moderate water deficiency still affects fiber development and overall quality (Hussain et al., 2020). Water availability is critical for sustaining cell turgor pressure and supporting carbohydrate assimilation in developing fibers (Abdelraheem et al., 2020; Kolahi et al., 2020). Expression of the sucrose synthase gene, essential for fiber elongation during the development, is downregulated under water deficiency stress, leading to decreased sucrose availability and shorter fiber lengths (Ahmed et al., 2018; Gao et al., 2020; Loka et al., 2020). Water deficiency during the reproductive stage also reduces fiber length and fiber density per seed, while increasing fiber thickness, ultimately lowering the fiber quality index (Hu et al., 2018). Wang et al. (2016) reported a linear decline in fiber length and strength with decreasing leaf water potential, suggesting its utility as an indicator of fiber quality under water deficiency conditions. Snowden et al. (2014) further noted that full bloom water deficiency exerts a greater negative effect on fiber length and fineness compared to early bloom stress. During elongation, reduced sucrose content and diminished synthase activity lower turgor pressure, limiting final fiber length. The expression of sucrose synthase genes, together with stress-related defense pathway genes, plays a vital role in fiber development. Under water-deficit conditions, changes in the expression levels of these genes significantly influence fiber growth and associated traits (Ruan et al., 2003; Padmalatha et al., 2012; Kim et al., 2013; Ul-Allah et al., 2021; Zhu et al., 2024). Water deficiency during flowering also impairs fiber strength and increases the proportion of short fibers by disrupting cell expansion and cellulose deposition (Abdelraheem et al., 2020). Transcriptomic analysis by Padmalatha et al. (2012) indicates that fiber elongation is particularly affected, as genes involved in cell wall loosening and expansion are significantly downregulated under water deficiency conditions.

Ginning, the process of separating lint from seed, is essential for transforming raw cotton into usable fiber. Seed cotton moisture content during ginning affects fiber integrity. Optimal ginning occurs when seed cotton moisture is maintained between 5–6% (Hughs et al., 2004; Hardin et al., 2018). Maintaining cotton moisture levels above 6% during ginning is important for preserving fiber quality (Byler, 2006). Moisture imbalances require corrective measures, such as pre-ginning drying or moisture restoration which increase energy demands and operational costs (Hardin et al., 2018). Reduced ginning energy use offers clear advantages for both profitability and sustainability in the cotton industry which directly decrease processing costs. Ginning energy and rate are closely linked to traits such as fuzz percentage, fiber strength, length, and fineness, as well as boll weight and fiber density per seed (Bechere et al., 2011). However, the direct effects of water deficiency on ginning energy consumption and rate remain poorly characterized, presenting a critical research gap.

In addition to fiber, cottonseed represents an economically important byproduct, valued for its oil and high-protein meal (Zhuang et al., 2023). Typically, cottonseed contains 15–20% oil and 20–30% protein (Bellaloui and Turley, 2013; He et al., 2014). While seed composition is largely governed by genetic factors, environmental variables, particularly water availability, also play a significant role (Pettigrew and Dowd, 2014). Water deficiency may alter oil and protein content, although the interactive effects of irrigated management and genotype on seed quality remain insufficiently understood (Dowd et al., 2010). Such changes can significantly affect the economic value of cottonseed.

This study aims to assess the impact of water deficiency stress on three critical aspects of cotton production: ginning efficiency, fiber quality, and seed composition. Specifically, we compare irrigated and non-irrigated cultivation systems in 10 genetic varieties to: quantify differences in ginning efficiency with a focus on energy consumption and ginning rate under water deficiency stress; evaluate fiber traits including length, strength, fineness, and short fiber content under stress; and analyze seed composition, with an emphasis on oil and protein content in response to the stress. By addressing these objectives, the study seeks to inform strategies for improving cotton productivity and value under water deficiency conditions, enhancing both fiber processing efficiency and seed quality.

## Materials and methods

### Field design and plant materials

A total of ten cotton genotypes including MD 10-5, Reba P288, MD 52ne, 8327, 1517-99, 84524, 82514, MD 25-26ne, MD 15, and CIM 432 were evaluated under both irrigated and non-irrigated (water deficiency) conditions. MD 52ne (PI 634930) (Meredith, 2005), MD 15 (PI 642769) (Meredith, 2006), MD 25-26ne (PI 666042) (Meredith, 2013), and MD 10-5 (PI 675077) (Zeng et al., 2016) are *Gossypium hirsutum* L. from USDA, Stoneville, MS. Acala 1517-99 (1517-99) (PI 612326) is *Gossypium hirsutum* L. from New Mexico Agricultural Experiment Station (NMAES) (Cantrell et al., 2000). Reba P288 (PI 607158) is a triple hybrid involving *Gossypium hirsutum*, *Gossypium arboreum* and *Gossypium raimondii* from Paraguay (Alvarez et al., 1992). CIM 432 is from Pakistan that is developed to be highly heat tolerance. Three cultivars of 8327, 84524 and 82514 are unknown collections from USDA, Stoneville, MS.

The experiment followed a randomized complete block design with three replications per genotype in both irrigated and non-irrigated (water deficiency) fields. The study was conducted on Bosket fine sandy loam soil, a highly productive, well-drained, and deep soil type. Each experimental unit consisted of a single row plot, 42 feet in length, with 10 plots per row and 40 inches of spacing between rows. Plots were planted on May 1, 2024. In the irrigated treatment, plots received three times irrigated with 76.2 mm (3 in) of water applied each time with poly-pipe in furrow on June 26, July 17 and August 12, 2024. Standard insect and weed management practices were applied as needed throughout the growing season. Fertilization included applications of 134 kg/ha of K_2_O and 112 kg/ha of nitrogen at the first square stage. Weed control prior to planting involved pre-plant incorporation of Prowl (1.8 l/ha) and Valor (1.5 l/ha). Pre-emergence herbicides included Dual Magnum (0.6 l/ha; Syngenta Crop Protection, LLC., Greensboro, NC, USA) and STAPLE LX (0.1 l/ha; DuPont, Wilmington, DE). Insect control for thrips included application of Radiant at 0.1 l/ha. Defoliation and boll opening were facilitated with two applications of GINSTAR (thidiazuron and diuron; Bayer CropScience, NC, USA)—first at 0.2 l/ha and then at 0.4 l/ha—followed by SUPER BOLL (ethephon; DuPont, Wilmington, DE, USA) at 1.5 l/ha in October 2024.

We measured the height of 30–40 individuals at 80 day-after -germination (DAGs) in July and at 105 DAGs in August when we observed the bolls opened in the non-irrigated drought condition fields, while the irrigated fields are flowering and developing green bolls. Total bolls number per plant of 12 individuals for each genotype in both irrigated and non-irrigated fields were counted in October after defoliation. At the end of October, 50 open bolls per plot were randomly hand-harvested, with one boll taken per plant. These samples were used to assess ginning characteristics such as net ginning energy, ginning rate, fuzz percentage, and other fiber quality parameters. Seed cotton was weighed prior to ginning, and lint was weighed post-ginning to calculate lint turnout. Lint and fuzz percentage were calculated using the following formulas:


Lint%=[(fiber seed weight–fuzzy seed weight)/fiber seed weight]×100

Fuzz%=[(fuzzy seed weight–delinted seed weight)/fuzzy seed weight]×100

Seed index is the weight of 100 fuzzy seeds (g)

### Soil moisture measurement and precipitation monitor

The field experiment site, 2.1 ha, was divided into two sections, one for the water deficiency treatment (non-irrigated) and the other for the common furrow irrigated practiced in the Mississippi Delta region. Eight rows were planted between the two sections to limit the potential influence of irrigated water overflow into the non-irrigated zone. Soil moisture was measured using a PR Profile Probe soil moisture sensor in 10 cm depth increments to 40 cm depth (Dynamax, Houston, TX, USA). This was achieved by installing 32 access tubes, 16 each in irrigated and non-irrigated plots, using a tailor-made auger to ensure optimal soil-access tube contact for a snug fit into the augured holes. Volumetric soil moisture readings were taken once every week from May 30 to August 28, 2024. The volumetric soil moisture content (percentage) was converted to depth (millimeters) of soil water content using associated soil bulk densities at each depth level for every reading. Soil bulk densities corresponding to the 32 moisture logging plots were measured by collecting soil cores to 40 cm depth in 10 cm increments using a tractor-mounted Giddings hydraulic coring machine (Giddings Machine Company, Windsor, CO, USA). A soil probe with an inner diameter of 5 cm was used to core into the soil profile and collect bulk density samples. The following conversion formula was used in computing soil moisture content depth (SMC) in millimeters (mm):


$$\text{SMC }\left(\text{mm}\right)=\text{SM }\left(\text{vol}\right)\times \text{Soil Depth }\left(\text{cm}\right)\times \text{SoilBulkDensity }\left(\text{g}/{\text{cm}}^{3}\right)$$

where SM (vol) = volumetric water content (%), soil bulk density (grams per cubic centimeter), and soil depth in centimeters (cm).

We monitored weather and precipitation data at the Stoneville Experimental Station, MS (Elevation: 127 ft; Latitude: 33.4311° N; Longitude: 90.9108° W; Station ID: USC00228445) using records from the National Centers for Environmental Information (https://www.ncei.noaa.gov/access/past-weather/). In May 2024, the daily average high temperature was 84°F and the low was 64°F, with a total precipitation of 26.42 mm. In June, temperatures rose to an average high of 95°F and low of 72°F, with no precipitation. July averaged 89°F high and 73°F low, with 4.32 mm of precipitation. August saw the highest average temperatures, with a high of 97°F and low of 73°F, and again no precipitation was recorded. From May 1 to August 28, 2024, each irrigated (control) plot received a total of 25.93 cm of water, while non-irrigated (water deficiency) plots received only 3.07 cm from rainfall (**Supplementary Table 1**). We also monitored water deficiency conditions via the U.S. Drought Monitor (https://droughtmonitor.unl.edu) (**Supplementary Table 1**). In May 2024, conditions were categorized as “None” (normal). Starting in early June, the classification escalated to D0 (abnormally dry), progressing to D1 (moderate drought) by June 18. Between July 16 and July 23, the area experienced D3 (extreme drought). Conditions slightly improved in early August to D1, then worsened to D2 (severe drought) by mid-August, and returned to D3 by August 20 (**Supplementary Table 1**).

### Ginning efficiency test and data collection

The cotton used for the ginning efficiency studies was preconditioned in a humidity chamber at 55% relative humidity for three days prior to ginning. This was done to ensure that all the samples had similar moisture content such that moisture variation would not affect energy or fiber quality. Due to the limited quantity of hand-harvested test cotton, it was not feasible to use these samples for moisture content analysis. Instead, a commercial cultivar of upland cotton (*Gossypium hirsutum*) was simultaneously conditioned and used both for daily moisture testing and to monitor ginning energy variation across the ginning period.

Each day of ginning, three randomly selected samples of the conditioned commercial cotton were tested for moisture content using the standard oven-drying method (Shepherd, 1972). Concurrently, the experimental cotton samples scheduled for ginning that day were sealed in containers while still in the humidity chamber to preserve moisture content and prevent exposure to ambient environmental conditions. These containers remained sealed until immediately before ginning. Ginning was conducted over two consecutive days. At the beginning of each day, a power meter was activated to continuously record all energy use events. Even during non-ginning intervals—such as equipment cleanouts—the power meter remained active, allowing precise identification of individual ginning runs based on intervals of zero power consumption.

For each sample, the gin stand was idled for at least one minute prior to ginning. Ginning was performed using the same configuration described in Donohoe et al. (2023a), which included a conveyor belt feed system and a single operator manually loading each sample. Upon completion, as judged by the operator, the gin stand was allowed to idle for approximately one additional minute before being shut down. To monitor daily variation, at least three commercial cotton samples were ginned at random intervals each day for comparison. Immediately after each ginning run, seeds and lint were separately collected and weighed. To minimize cross-contamination between samples, the lint collection chamber was vacuumed thoroughly after each run. Power data collected during ginning were post-processed using an automated analysis method detailed by Donohoe et al. (2023B). This method identifies active ginning periods, integrates power measurements over time, and subtracts the energy associated with idle operation to determine net (active) ginning energy. Statistical analyses of ginning energy were performed at a significance level of α = 0.05.

### Fiber quality measurement

Prior to testing the lint samples, all samples were conditioned in the testing laboratory to bring the moisture content of the samples into equilibrium with the atmosphere of the lab. The HVI 1000 instrument (Uster Technologies, Switzerland) was used to assess the lint samples collected during ginning. Each lint sample was subsampled and tested five times to address within-sample variability and improve measurement accuracy. Several key fiber traits were evaluated, including micronaire (MIC), an indirect measure of both fineness and maturity with higher values generally indicating coarser and more mature fibers; upper half mean length (UHML) indicates the average length of the longest 50% of fibers in the sample; fiber uniformity index (UI), calculated as the ratio of mean fiber length to UHML, representing the uniformity of fiber length distribution; and fiber strength (STR), expressed in grams per tex (g tex^-^¹), was measured as the force required to break a fiber bundle, indicating tensile strength. All measurements were conducted in accordance with standard procedures for testing cotton fibers to ensure reliability and comparability of results (https://www.cottoninc.com/wp-content/uploads/2017/02/Classification-of-Cotton.pdf; https://www.astm.org/).

### Cottonseed protein, oil and fiber analysis

Mature, delinted cottonseeds were ground into a fine powder using a Laboratory Mill 3600 (Perten Instruments, Springfield, IL, USA) to prepare samples for compositional analysis. Protein and oil contents were quantified using a DS3-F Near-Infrared Reflectance Spectrometer (FOSS North America, Inc., Eden Prairie, MN, USA) equipped with ISIscan™ Nova operating software. The instrument utilized dual detectors—Silicon (850–1100 nm) and Lead Sulfide (1100–2500 nm)—to capture spectral data across a wavelength range of 850 to 2500 nm. Seven subsamples were scanned per sample to ensure accuracy and reproducibility. Protein (%), oil (%) and fiber (%) were calculated on a dry-weight basis.

### Statistical analysis

A paired t-test was used to determine if there’s a significant difference between non-irrigated (water deficiency) and irrigated (control) on plant height, total boll number per plant, ginning rate, ginning energy, fiber traits and cottonseed composition denoted by p-values (p< 0.05*, p< 0.01**, p< 0.001***). Statistical analyses of all measured traits associated with the cotton genotypes were conducted using the plot data and then subjected to an analysis of variance (ANOVA) using SAS 9.3 (SAS Institute Inc., Cary, NC, USA). PROC GLM statistical procedure was used to determine the statistical significance of various sources of variation. The least significant difference (LSD) at the 5% level was used for mean separations. The objective was to evaluate ginning rate, ginning energy, fiber quality traits (measured via HVI), and seed composition traits (analyzed via NIR spectroscopy). Additionally, Analysis of Means (ANOM) charts were generated to visually assess decision thresholds and identify statistically significant deviations from the grand mean under both treatments. Graphical analysis and simultaneous comparison of genotype performance under irrigated and non-irrigated were conducted using SigmaXL software. To explore interrelationships among traits, Pearson’s correlation coefficients were calculated using the R statistical environment (R Core Team), providing insights into trait associations under varying environmental conditions.

## Results

### Effect of water deficiency on plant height and the number of bolls per plant

**Figure 1:**
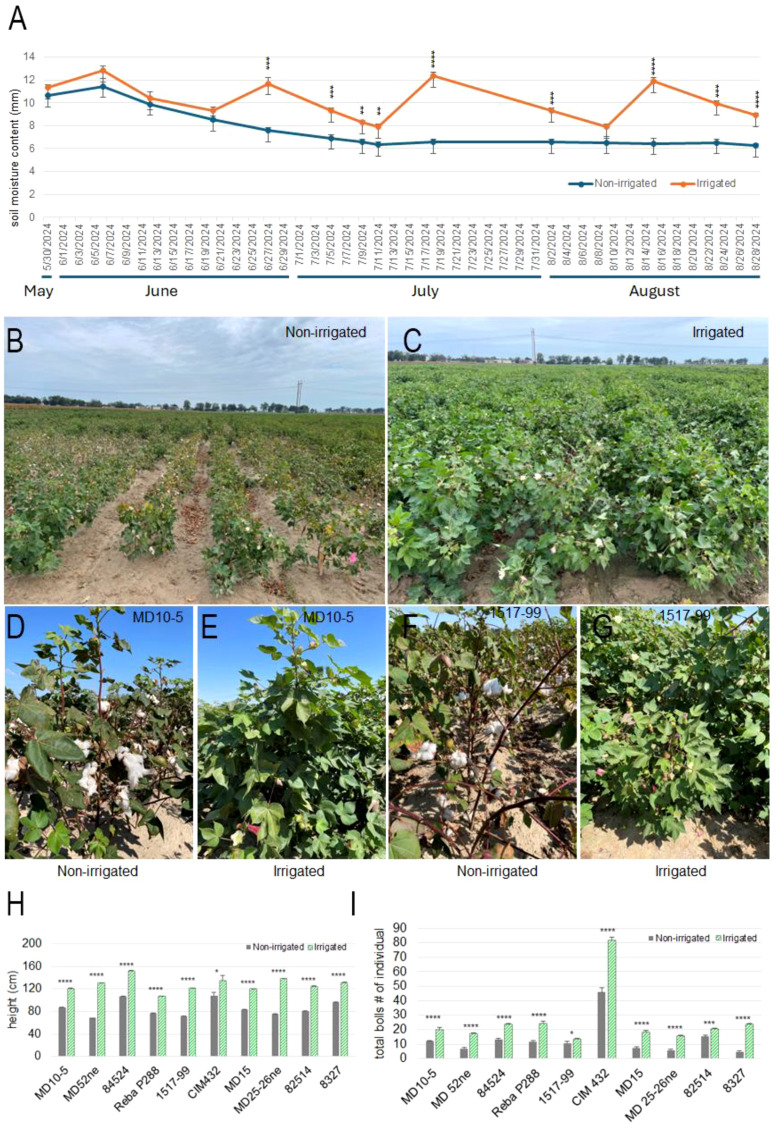
Soil moisture content 0–40 cm depth from May to August and cotton growth under non-irrigated (water deficiency) and irrigated conditions in late August, and harvested in October. **(A)** Soil moisture content (mm) from May to August, with blue lines representing non-irrigated (water deficiency) and orange lines representing irrigated conditions. Data points are means with error bars indicating standard deviation. **(B)** Field view of non-irrigated cotton plants in late August, showing plant growth under water deficiency stress. **(C)** Field view of irrigated cotton plants in late August, showing plant healthy growth. **(D)** Non-irrigated MD 10–5 genotype, displaying limited growth and opening boll. **(E)** Irrigated MD 10–5 genotype, showing robust growth and boll production. **(F)** Non-irrigated 1517–99 genotype, with reduced foliage and opening boll. **(G)** Irrigated 1517–99 genotype, exhibiting vigorous growth and increased boll production. **(H)** Plant height (cm) across 10 cotton genotypes under water deficiency (gray bars) and irrigated (green bars) conditions in late August (105 DAG). Bars represent means with error bars indicating standard deviation. **(I)** Number of total bolls per plant across the same genotypes under non-irrigated (gray bars) and irrigated (green bars) conditions in October when harvested. Bars represent means with error bars indicating standard deviation. Genotypes tested include MD 10-5, MD 25-26ne, 84524, Reba P288, 1517-99, CIM 432, MD 15, MD 52ne, 82514, and 8327. Statistical significance between non-irrigated and irrigated conditions is denoted by p-values above each genotype pair (p< 0.05*, p< 0.01**, p< 0.001***, p< 0.0001****).

We monitored weather, precipitation data and drought conditions at the Stoneville Experimental Station, MS using records from the National Centers for Environmental Information (https://www.ncei.noaa.gov/access/past-weather/) and the U.S. Drought Monitor (https://droughtmonitor.unl.edu/Maps/). From May 1 to August 28, 2024, each irrigated plot received a total of 10.21 inches of water, while non-irrigated (water deficiency) plots received only 1.21 inches from rainfall. From the mid of June to the end of August, the area experienced severe drought **Supplementary Table 1**). The average soil moisture content (SMC) was measured and calculated in 10 cm depth increments to 40 cm depth over time for two treatments “irrigated” and “non-irrigated” from May 30, 2024, to August 28, 2024 (**Figure 1A**; **Supplementary Figure 1**). Initially, both treatment fields exhibited similar SMC levels, with the irrigated field showing slightly higher values. Consistent with the weather and drought records, the irrigated field consistently maintained higher SMC compared to the non-irrigated field over the duration of the study. The non-irrigated treatment displayed a gradual decline in SMC, with only minor fluctuations and a noticeably lower overall trend. This difference became particularly pronounced from late June through late August, during which the 0–40 cm depth SMC in the non-irrigated group ranged from 6.23 mm to 7.59 mm, significantly lower than the irrigated field, which ranged from 7.89 mm to 12.36 mm (**Figure 1A**). Under non-irrigated conditions (water deficiency), cotton plants exhibited floral abortion, boll shedding, and early senescence of both leaves and bolls. While irrigated plants across all genotypes continued to flower and remained green at 105 days after germination (DAG) (**Figures 1C, E, G**), water deficiency plants showed accelerated boll opening and maturation (**Figures 1B, D, F**).

Water deficiency stress caused a significant reduction in plant height at both 80 and 105 DAG for all genotypes. Plants grown under irrigated conditions consistently exhibited greater height, indicative of improved vegetative growth. At 80 DAG, notable reductions in height were observed in genotypes MD 52ne (39.75%), MD 25-26ne (39.45%), and 1517-99 (27.16%) when compared to their irrigated counterparts. These genotypes, although performing well under control conditions, showed marked sensitivity to water deficiency. In contrast, MD 10-5, Reba P288, 8327, and CIM 432 exhibited comparatively smaller reductions of 13.78%, 15.60%, 16.60%, and 17.80%, respectively (**Supplementary Figure 2**). By 105 DAG, the impact of stress was more pronounced, with MD 52ne, MD 25-26ne, and 1517–99 again displaying substantial declines in height—47.72%, 45.87%, and 40.95%, respectively, suggesting consistent water deficiency sensitivity. Meanwhile, MD 10-5, Reba P288, 8327, and CIM 432 showed reduced sensitivity, with declines of 28.30%, 28.13%, 26.63%, and 19.71%, respectively. Among all genotypes, CIM 432 demonstrated the least reduction in plant height across both developmental stages, indicating enhanced water deficiency tolerance. These differences were statistically significant in most cases, underscoring the severity and consistency of water deficiency effects across time points. Overall, genotypes such as MD 52ne, MD 25-26ne, and 1517–99 appeared to be the most water deficiency-sensitive, whereas CIM 432 showed comparatively greater tolerance as measured by vegetative development (**Figure 1H**).

In terms of reproductive development, non-irrigated also led to a significant reduction in the number of total bolls per plant in most genotypes by October when the plants were harvested (**Figure 1I**). However, the magnitude of this reduction varied. Genotypes 8327, MD 25-26ne, MD 15, and MD 52ne exhibited the most substantial declines in boll number under water deficiency: 80.0%, 61.90%, 60.81%, and 59.42%, respectively, relative to the irrigated condition. Genotypes such as 8327 and 1517–99 also showed considerable reductions of 25.6% and 23.7%, respectively, indicating moderately stable performance under water deficiency (**Figure 1I**). Interestingly, CIM 432, although showing a 43.55% reduction in boll number compared to irrigated plots, still maintained the highest absolute number of bolls under stress conditions. While most genotypes averaged 16–23 bolls per plant under irrigated, CIM 432 produced an average of 46 bolls under water deficiency and 82 under irrigated. This exceptional performance suggests that despite some sensitivity during reproductive growth, CIM 432 maintains higher productivity under water-limited conditions and thus represents a promising candidate for cultivation in water deficit environments.

### Effect of water supply during cotton growth on ginning energy and ginning rate

The wet basis average moisture content of the commercial cotton samples tested was 7.7% and 8.2% on the first and second day of the experiment, respectively. A t-test revealed no statistically significant difference between the two days (p = 0.189). The net (active) energy consumption during ginning of the commercial control cotton averaged 4.65 Wh/kg on the first day and 4.35 Wh/kg on the second. This difference was not statistically significant (p = 0.055), suggesting consistent ginning energy requirements across both days.

**Figure 2:**
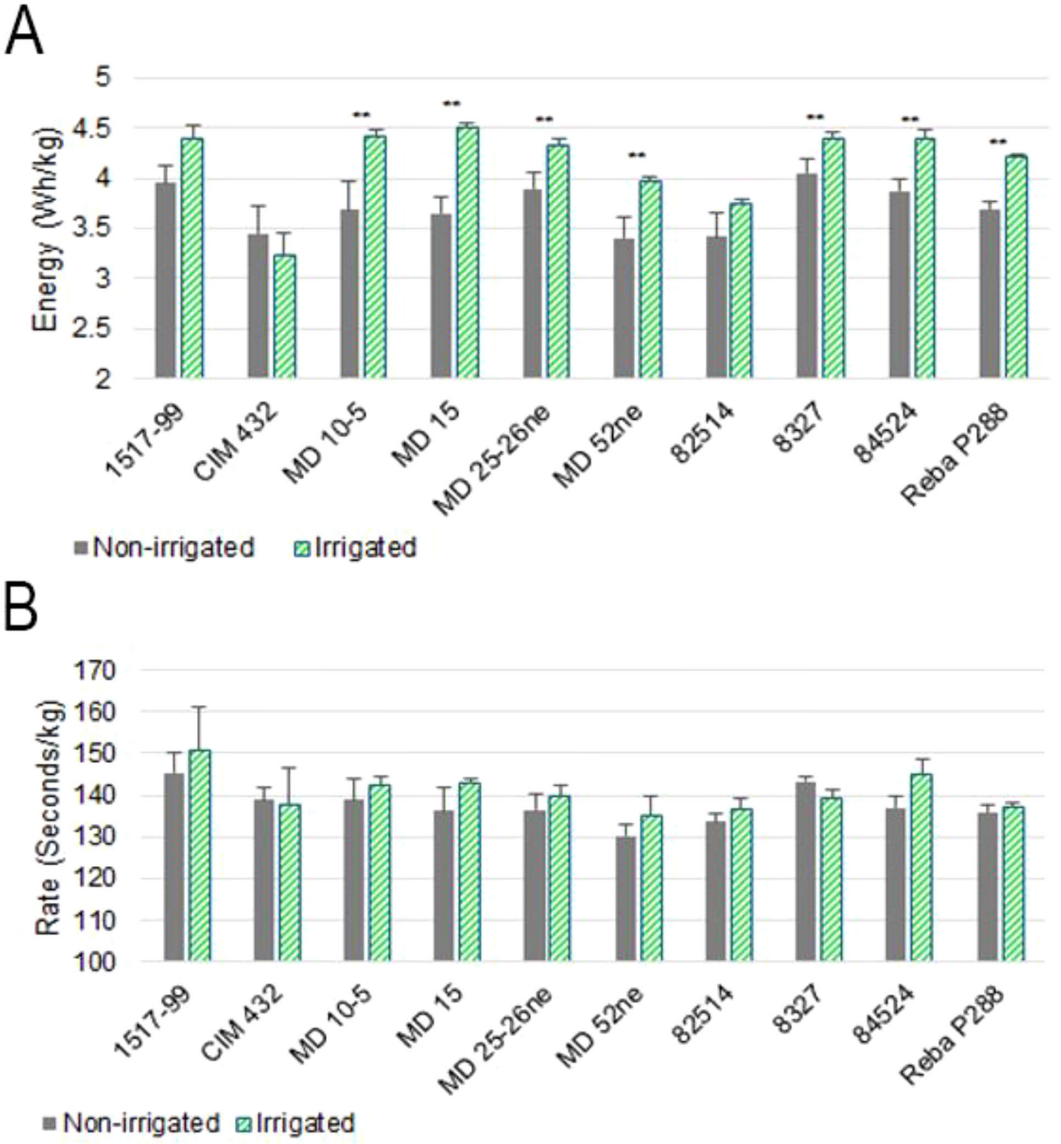
Cotton active (net) ginning energy and ginning rate across 10 cotton genotypes under non-irrigated and irrigated conditions. **(A)** Active (net) ginning energy (Wh/kg) for genotypes 1517-99, CIM 432, MD 10-5, MD 15, MD 25-26ne, MD 52ne, 82514, 8327, 84524, and Reba P288. Bars represent mean values with error bars indicating standard deviation. Statistical significance between non-irrigated and irrigated is denoted by p-values above each genotype pair. **(B)** Ginning rate (seconds/kg) for the same genotypes. Bars represent mean values with error bars indicating standard deviation. No significant differences were observed between non-irrigated and irrigated treatments for ginning rate across all genotypes (p > 0.05). Green bars represent irrigated conditions, and gray bars represent non-irrigated conditions.

**Figure 2A** presents the net ginning energy required for each cotton genotype under both water deficiency and irrigated control conditions. Significant differences in ginning energy were observed in seven genotypes: MD10-5, MD 15, MD 25-26ne, 8327, 84524, Reba P288, and MD 52ne. In all cases where differences were statistically significant, cotton grown under water deficiency conditions required less energy to gin compared to the irrigated controls. An exception was observed in CIM 432, where the water deficiency-grown cotton exhibited a slightly higher average ginning energy than the control; however, this difference was not statistically significant. These findings suggest that water deficiency can influence ginning energy requirements in a genotype-dependent manner. Importantly, no significant differences in ginning rate were found across any genotype or treatment, indicating that water deficit stress had a minimal impact on the speed of fiber separation (**Figure 2B**). Among the genotypes, MD 15, MD10-5, and MD 52ne exhibited the most notable reductions in ginning energy under water deficiency conditions, implying that ginning efficiency in these lines is particularly sensitive to water deficiency stress. Conversely, genotypes such as CIM 432, 1517-99, and 82514 showed no significant changes in ginning energy or efficiency across both treatments, suggesting greater stability or water deficiency tolerance in terms of fiber processing characteristics.

### Impact of water deficiency on fiber quality

**Figure 3:**
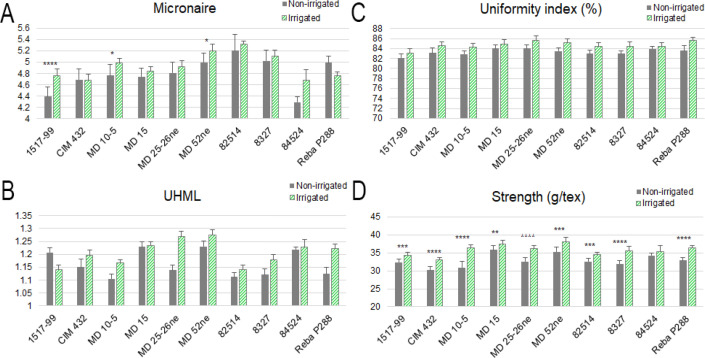
Analysis of four High Volume Instrument (HVI) fiber quality parameters across 10 cotton genotypes (1517-99, CIM 432, MD 10-5, MD 15, MD 25-26ne, MD 52ne, 82514, 8327, 84524, and Reba P288) under non-irrigated and irrigated conditions. Each panel represents a different fiber quality parameter: Micronaire **(A)**; Upper Half Mean Length (UHML, inch) **(B)**; Uniformity Index (%) **(C)**; Strength (g/tex) **(D)**. Bars represent mean values with error bars indicating standard deviation. Green bars represent irrigated conditions, and gray bars represent non-irrigated (water deficiency) conditions. Statistical significance between non-irrigated and irrigated conditions is denoted by p-values above each genotype pair (p< 0.05*, p< 0.01**, p< 0.001***, p< 0.0001****).

An analysis of several High Volume Instrument (HVI) fiber quality parameters across 10 cotton genotypes under both water deficiency and irrigated conditions revealed several significant trends (**Figure 3**). Among these parameters, micronaire (MIC) values showed fewer statistically significant differences overall, indicating general trait stability. However, 1517-99, 84524, and Reba P288 exhibited significant changes, suggesting a greater sensitivity to water deficit in these lines (**Figure 3A**). Upper Half Mean Length (UHML) was significantly reduced under water deficiency stress in MD10-5, MD 25-26ne, Reba P288, and CIM 432, indicating these genotypes are particularly sensitive to water limitation. In contrast, 1517–99 and 84524 maintained a relatively stable UHML, suggesting greater water deficiency tolerance for this trait (**Figure 3B**). Uniformity Index (UI) declined significantly in genotypes MD10-5, MD 52ne, 8327, and Reba P288 under water deficiency conditions. Genotypes 1517–99 and CIM 432 exhibited moderate reductions, while MD 15 and 84524 showed no significant change, suggesting better stability in fiber uniformity under stress (**Figure 3C**). Strength (STR) declined significantly in all genotypes under water deficiency stress, with the sole exception of 84524, which maintained strength levels comparable to irrigated conditions. This suggests 84524 may carry valuable genetic traits for fiber strength retention under stress (**Figure 3D**). Overall, water deficiency stress negatively impacted key fiber quality parameters, including UHML, UI, and STR. In contrast, MIC was more stable across conditions. Notably, genotypes CIM 432, MD 15 and 84524 displayed the most consistent fiber quality performance under both non-irrigated and irrigated, positioning them as promising candidates for breeding programs aimed at improving water deficiency tolerance without compromising fiber quality.

### Protein, oil and fiber contents in cottonseeds

**Figure 4:**
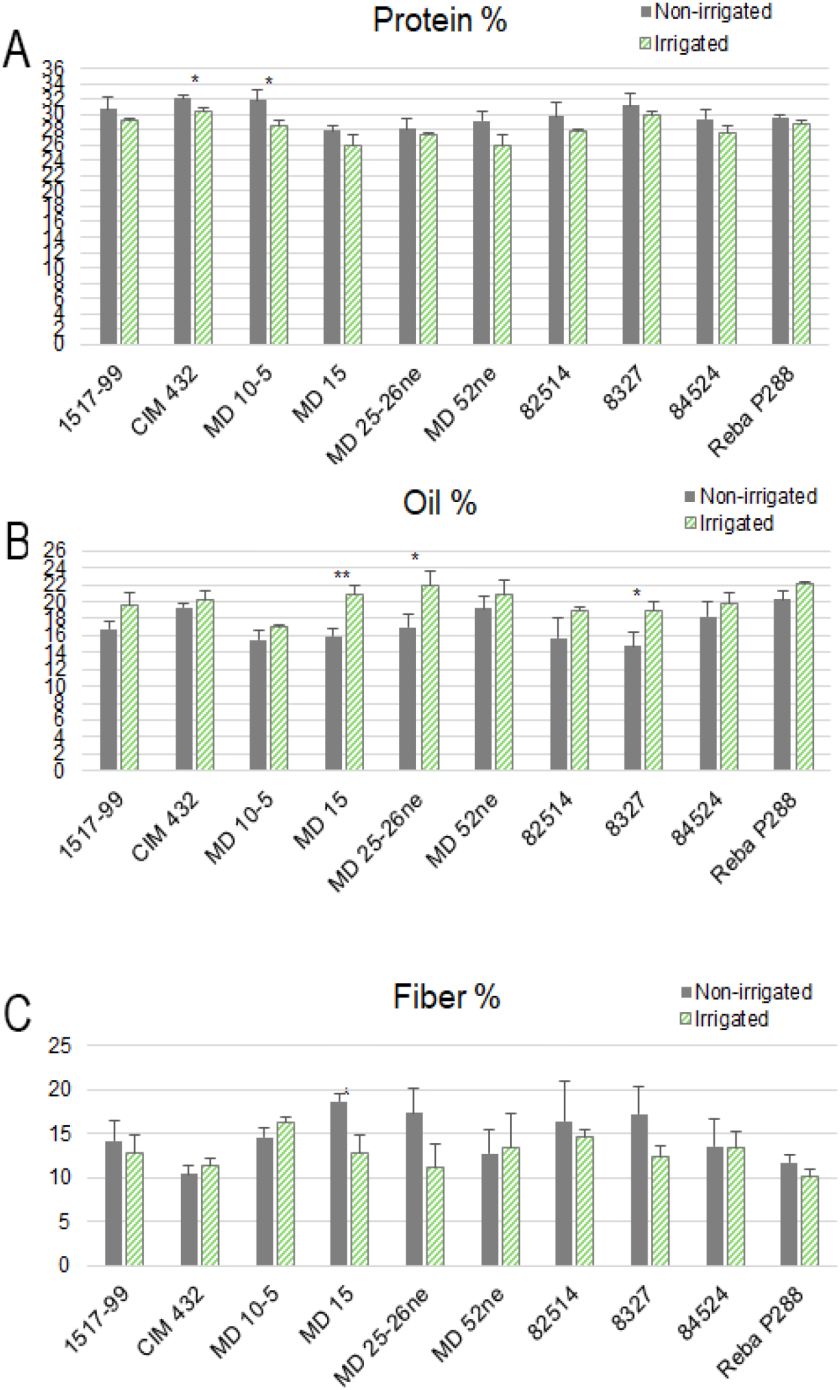
Near-Infrared Reflectance (NIR) analysis of cottonseed composition on a dry weight basis across 10 cotton genotypes under non-irrigated and irrigated conditions. Genotypes tested include 1517-99, CIM 432, MD 10-5, MD 15, MD 25-26ne, MD 52ne, 82514, 8327, 84524, and Reba P288. Each panel represents a different compositional parameter: **(A)** Protein content (%); **(B)** Oil content (%); **(C)** Fiber content (%). Bars represent mean values with error bars indicating standard deviation. Green bars represent control conditions, and gray bars represent water deficiency conditions. Statistical significance between non-irrigated and irrigated treatments is denoted by p-values above each genotype pair (p< 0.05*, p< 0.01**).

Under irrigated conditions, cottonseed protein content ranged from 25.94% to 30.37%, with the highest value recorded in genotype CIM 432 and the lowest in MD 52ne. Under non-irrigated condition, protein content ranged from 27.83% to 32.11%, again with CIM 432 exhibiting the highest value and MD 15 the lowest (**Figure 4A**). These results indicate that CIM 432 consistently maintains high protein content across moisture regimes, while MD 15 appears more sensitive to water deficiency stress.

For oil content under irrigated, values ranged from 17.02% to 22.04%. Reba P288 exhibited the highest oil percentage, while MD 10–5 had the lowest (**Figure 4B**). Under water deficiency, cottonseed oil content decreased overall, ranging from 14.73% to 20.22%. Reba P288 again had the highest oil content, whereas 8327 recorded the lowest, highlighting a genotype-specific response to the water deficit. The response of oil content to irrigated varied significantly among genotypes. Statistically significant reductions in oil content under stress were observed in MD 15, MD 25-26ne, and 8327. In contrast, other genotypes exhibited moderate or non-significant differences between irrigated and non-irrigated conditions. Compared to oil, protein and seed fiber content were less affected by irrigated treatment across the genotypes, showing relative stability under water deficiency stress (**Figure 4C**). The seed fiber content measured by NIR is different from the lint fiber quality that we analyzed by HVI.

### Correlation analysis between ginning efficiency and seed traits under irrigated and non-irrigated conditions

**Figure 5:**
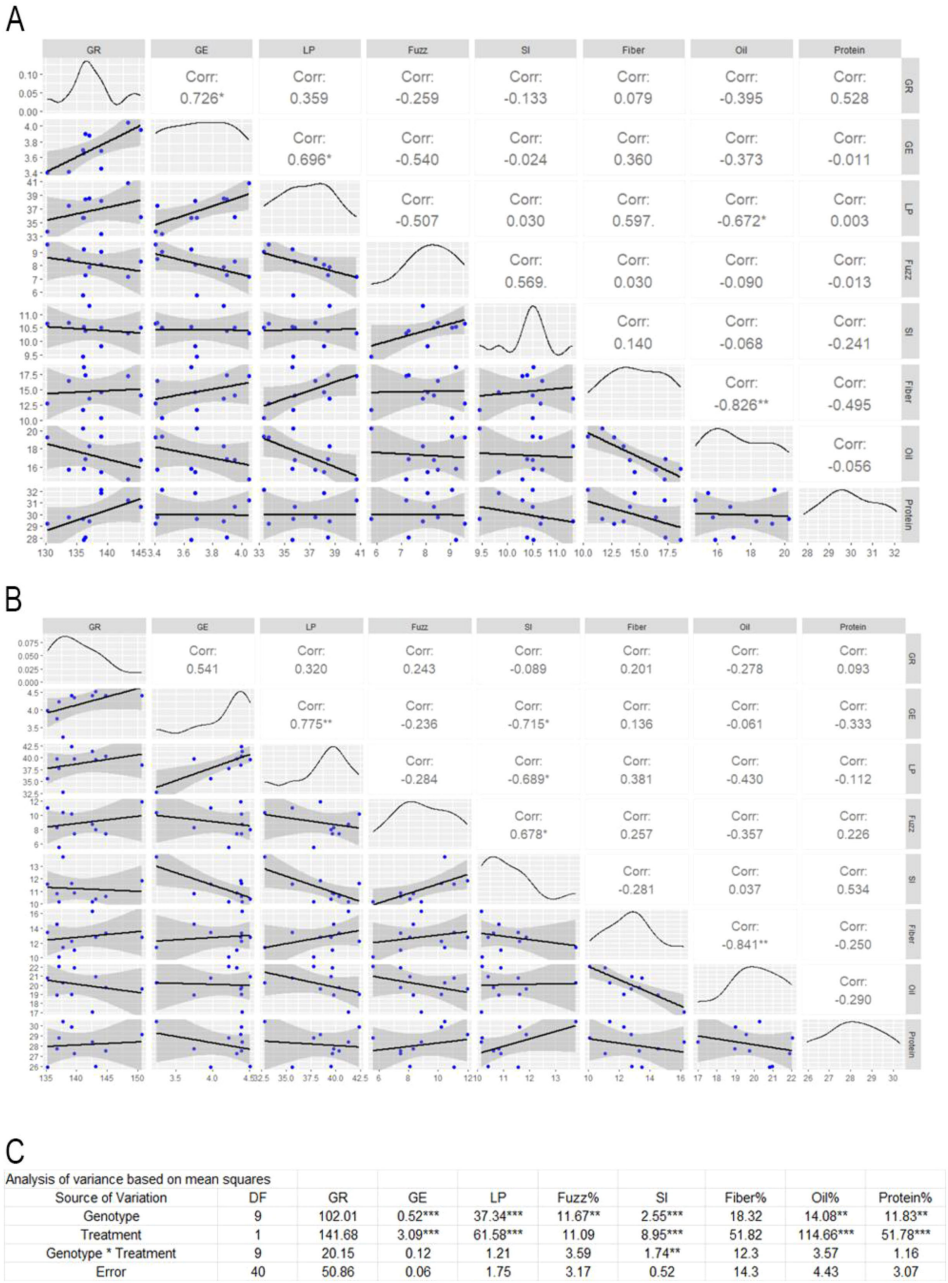
Correlation matrices and ANOVA analysis showing the relationships among cotton ginning energy, ginning rate, lint percentage, fuzz content and seed traits under different conditions. **(A)** Pairwise correlation matrix for fiber and seed traits under non-irrigated (water deficiency) treatment. Blue dots represent individual data points, with a fitted regression line and 95% confidence interval (gray shading). Pearson correlation coefficients (Corr) are reported for each pairwise comparison; asterisks indicate significance (*p*< 0.05). **(B)** Pairwise correlation matrix for the same traits under irrigated condition, presented as in panel **(A)**. **(C)** ANOVA summary table showing sources of variation for each trait, including Genotype, Treatment, and Genotype × Treatment interaction. Degrees of freedom (DF) and P-values are shown, with significance levels indicated as follows: p< 0.05*, p< 0.01**, p< 0.001***. Traits analyzed: GR (ginning rate), GE (ginning energy), LP (lint percentage), Fuzz%, SI, Fiber%, Oil%, and Protein%.

The correlation matrix plot illustrates the relationships between ginning rate (GR), ginning energy (GE), lint percentage (LP), fuzz content, seed index (SI), seed fiber content, oil content, and protein content under non-irrigated and irrigated conditions (**Figure 5**; **Supplementary Figure 3**). Under non-irrigated (water deficiency), GR and GE exhibited a strong positive correlation (r = 0.726, p< 0.05), indicating that increased GE is associated with higher GR (**Figure 5A**). GE also showed a positive correlation with LP (r = 0.696, p< 0.05). A moderate negative correlation was observed between GR and oil content (r = -0.395), though not statistically significant, potentially indicating a trade-off between ginning efficiency and oil accumulation. Fiber and oil content demonstrated a strong and significant negative correlation (r = -0.826, p< 0.01), implying that higher fiber yield may come at the expense of oil content under water deficiency. Other associations, such as GR and LP (r = 0.359), GR and protein (r = 0.528), and GE and protein (r = -0.011), were weak or not statistically significant. Similarly, moderate but non-significant trends were seen between SI and fiber % (r = 0.140) and LP and oil (r = -0.672). Several trait combinations showed negligible correlation, including GR with fuzz content (r = -0.259), GE with SI (r = -0.024), and most pairings involving protein content, which appeared largely independent of other traits under water deficiency conditions. These findings underscore the complex interplay between seed performance and compositional characteristics and highlight the importance of balancing multiple traits when selecting water deficiency-tolerance cotton genotypes.

Under irrigated conditions, a strong positive and statistically significant correlation between LP and GE (r = 0.775, p< 0.01), and a moderate positive association of GR with LP (r = 0.320) were observed suggesting that high lint percentage will need high ginning energy and ginning rate (**Figure 5B**). Conversely, GE had significant negative correlations with SI (r = -0.715, p< 0.05) hinting that small seeds may need more ginning energy. A notable finding was the strong negative correlation between lint fiber and oil content (r = -0.841, p< 0.01), reinforcing the inverse relationship observed under water deficiency conditions and pointing toward a trade-off between cottonseeds fiber yield and oil accumulation regardless of irrigated status. Most other trait correlations, including those involving protein, were weak or not statistically significant, suggesting limited dependency of protein content on the other measured parameters under control conditions. Overall, the pattern of relationships under irrigated emphasizes the interconnectedness of energy use, ginning rate, and fiber production, while again highlighting a critical negative link between fiber and oil content.

The ANOVA analysis revealed significant genotypic differences across most of the evaluated traits, including GE, LP, fuzz percentage, SI, oil content, and protein content (**Figure 5C**). However, no significant genotypic variation was observed for GR and seed fiber percentage. Treatment effects representing the impact of irrigated versus water deficiency were significant for all traits except GR, fuzz percentage, and fiber percentage, indicating a strong environmental influence on most seed and ginning traits. Notably, genotype-by-treatment interaction was only detected in SI (r = 1.74**), suggesting that the response of SI to environmental conditions varies across genotypes, while other traits exhibited more stable responses across different conditions. These findings underscore the importance of both genetic and environmental contributions to trait expression and highlight SI as particularly responsive to genotype-by-environment interactions.

### Correlation analysis of fiber quality under irrigated and non-irrigated conditions

**Figure 6:**
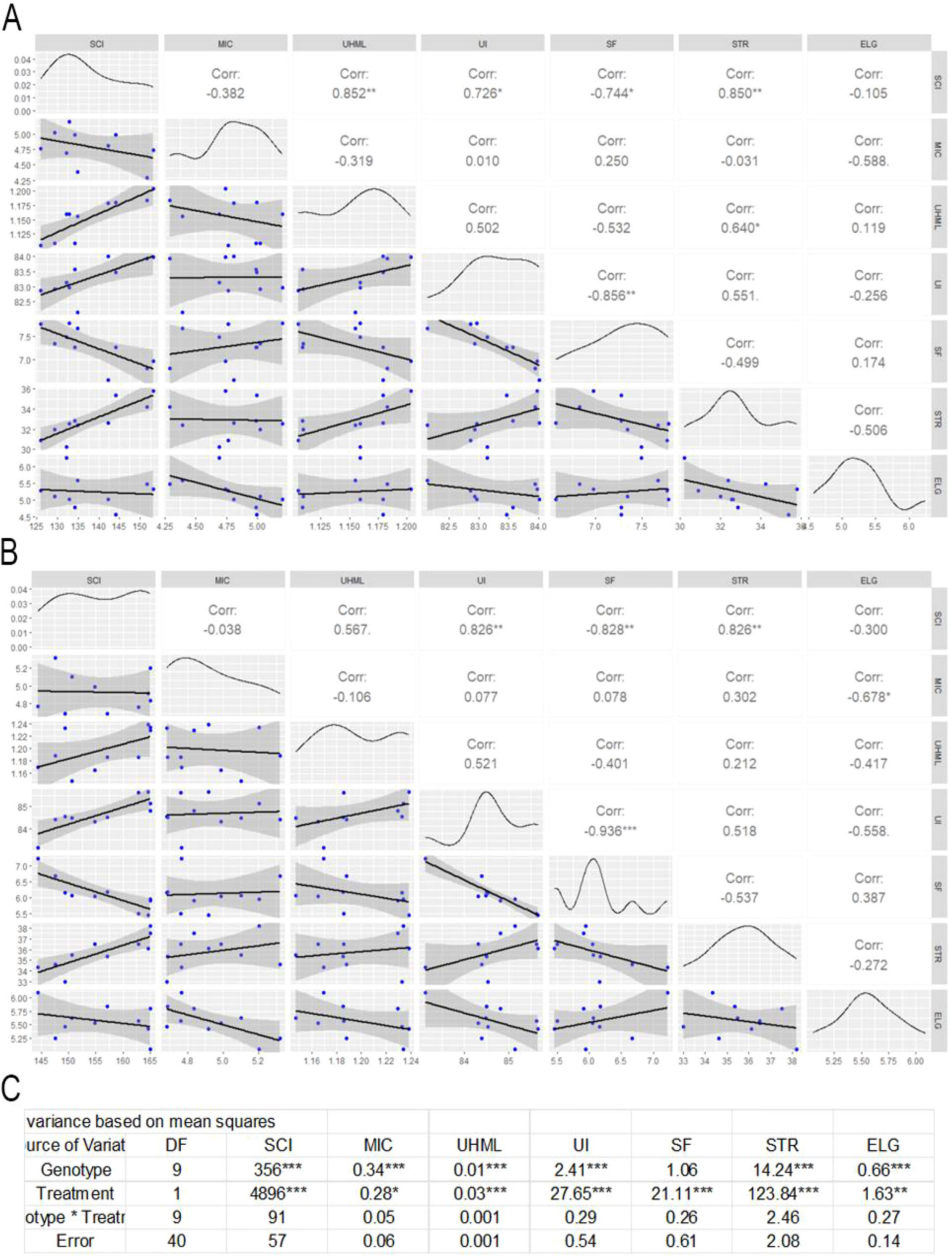
Correlation matrices and ANOVA results for fiber quality traits in cotton under irrigated and non-irrigated (water deficiency) conditions. **(A)** Pairwise correlation matrix of fiber quality traits under non-irrigated treatment. Each plot includes blue data points with a fitted regression line and 95% confidence interval (gray shading). Pearson correlation coefficients (Corr) are reported, with significance indicated by asterisks (p< 0.05*, p< 0.01**, p< 0.001***). **(B)** Correlation matrix for the same fiber quality traits under irrigated condition, following the same format as panel **(A)**. **(C)** ANOVA summary table showing the effects of Genotype, Treatment, and their interaction on each trait. Degrees of freedom (DF) and p-values are reported, with significance levels indicated: p< 0.05*, p< 0.01**, p< 0.001***. Traits include MIC (micronaire), UHML (upper half length), UI (uniformity index), SF (short fiber content), and STR (fiber strength).

The correlation plot for fiber traits under water deficiency conditions reveals significant relationships among several key parameters. Notably, a strong positive correlation exists between UHML and STR (r = 0.640*), as well as UHML and UI (r = 0.502), indicating that longer fibers tend to be stronger and more uniform under water deficiency stress. Conversely, SF shows a strong negative correlation with UI (r = -0.856**) suggesting that higher short fiber content is associated with lower fiber uniformity (**Figure 6A**). Overall, these results highlight the critical interdependence of fiber quality parameters under water deficiency conditions, where improvements in fiber length and uniformity are closely tied to reductions in short fiber content and increases in strength.

The correlation plot of fiber traits under irrigated conditions demonstrates several significant relationships among the fiber quality parameters. SF shows a very strong negative correlation with UI (r = -0.936***) and strong negative correlations with both STR (r = -0.537) and UHML (r = -0.401), reinforcing the idea that higher short fiber content negatively impacts fiber quality (**Figure 6B**). MIC has moderate correlation with UHML, UI and STR under both irrigated and non-irrigated. Overall, under optimal growth conditions, the interplay of these traits remains consistent with known fiber quality dynamics, emphasizing the critical importance of reducing short fibers to enhance overall fiber performance.

The ANOVA results for HVI fiber traits reveal significant genotypic differences for all evaluated traits except SFC, indicating substantial genetic variability among genotypes in most fiber quality attributes such as MIC, UHML, UI, and STR. Additionally, treatment effects were found to be highly significant for all traits, demonstrating the strong influence of environmental conditions on fiber quality (**Figure 6C**). However, no significant genotype-by-treatment interactions were detected across all traits, suggesting that genotypic performance was consistent across environmental conditions. This indicates that while both genotype and treatment independently affect fiber traits, the response of genotypes to different treatments is relatively stable, simplifying selection processes in breeding programs.

## Discussion

Water deficiency poses a significant constraint to cotton productivity, impacting both plant development and fiber quality (Saeed et al., 2024). This study evaluated ten cotton genotypes under irrigated and non-irrigated conditions to assess response to ginning efficiency, fiber quality traits, and seed composition. Water deficiency significantly hampered both vegetative and reproductive development in cotton, as demonstrated by reductions in plant height and boll production. Studies have shown that under water-deficient conditions, early senescence, floral abortion, and boll shedding are triggered as survival mechanisms—though ultimately detrimental to yield (Hou et al., 2024; Qureshi et al., 2021; Zafar et al., 2023). Our findings reveal considerable genotypic differences in response to water deficiency. Genotypes such as MD 52ne, MD 25-26ne, and 1517–99 experienced the steepest declines in plant height at 80 and 105 days after germination, highlighting their susceptibility to water deficiency. Conversely, CIM 432 and MD 10–5 displayed greater resilience, with CIM 432 notably outperforming all other genotypes in boll retention under stress. Although the non-irrigated plots showed a 43.55% reduction in boll production in CIM 432 compared to its irrigated counterpart, it still produced the highest number of bolls, making it a strong candidate for water deficit-resilient breeding. These observations support growing literature on the importance of genotypic selection for traits such as sustained vegetative growth and boll retention under water stress (Abdelraheem et al., 2021A; Saeed et al., 2024).

### Ginning efficiency and fiber quality under water deficiency

Improving the ginning efficiency benefits for both economic performance and environmental sustainability. Lower energy requirements reduce operational costs and enhance economic returns and support long-term sustainable production. Recent findings, along with prior research, highlight the intricate relationship between drought stress, cotton fiber quality, and ginning efficiency that carry significant implications for both agronomic management and breeding strategies (Byler, 2006; Abdelraheem et al., 2021b). However, genotype-specific reductions in ginning energy under water deficiency, particularly in MD 15, MD10-5, and MD 52ne, indicate physiological responses to water deficiency stress that likely altered fiber or seed coat characteristics, reducing the mechanical force required for fiber separation (Zafar et al., 2023). In contrast, CIM 432 showed a stable ginning energy under water deficiency, coupled with its favorable agronomic performance, position CIM 432 as a promising candidate for cultivation in water deficiency-prone regions.

The impact of water deficiency on fiber quality metrics further underscores the importance of genotypic variation in water deficiency tolerance (Sawan, 2018; Abdelraheem et al., 2021a). Declines in UI were particularly notable in genotypes such as MD10-5, MD 52ne, 8327, and Reba P288, indicating compromised fiber uniformity. Most genotypes exhibited increased Short Fiber Index (SFI) under non-irrigated conditions except 1517–99 raising concerns about spinning efficiency. Interestingly, micronaire remained relatively stable in most genotypes, except for 1517-99, 84524, and Reba P288. Significant reductions in fiber length and strength under water deficiency in genotypes like MD10-5, MD 25-26ne, and Reba P288 align with earlier studies linking water deficiency to disrupted cellulose synthesis and elongation processes (Tang et al., 2017; Zafar et al., 2023). However, 82514’s strong retention of fiber strength and CIM 432’s consistent performance across both ginning and fiber quality traits reinforces their potential as valuable genetic resources.

### Variation in cottonseed composition under water deficiency stress

Previous studies have documented mixed effects of irrigation on cottonseed oil and protein content, with responses varying from beneficial to negligible or even detrimental (Bertrand et al., 2005; Dowd et al., 2010; Pettigrew and Dowd, 2014; Bellaloui and Turley, 2013, Bellaloui et al., 2019; He et al., 2014, 2015; Kouser et al., 2015). These variable outcomes are likely driven by genotype-specific sensitivities to drought and heat, as well as complex genotype-by-environment interactions. In this context, CIM 432 stood out for its consistently high protein content reaching up to 32.11% even under water deficiency suggesting a resilient metabolic profile possibly linked to enhanced nitrogen assimilation, as observed in other water deficiency-tolerant genotypes (Zafar et al., 2023). In contrast, oil content proved more susceptible to water stress, with genotypes like MD 15, MD 25-26ne, and 8327 experiencing significant declines—down to 14.73% in 8327. This pattern reflects the greater vulnerability of lipid biosynthesis to water deficit, likely due to impaired carbon partitioning toward storage compounds (Sawan, 2018). Interestingly, Reba P288 maintained high oil levels across both conditions (up to 22.04% when irrigated), suggesting it harbors genetic traits favorable for oil stability under stress. These observations support the need for genotype-specific breeding strategies aimed at improving seed composition under water deficiency conditions.

The observed stability of protein content across both irrigated and non-irrigated conditions, coupled with its lack of significant correlation with oil and seed fiber traits, implies that protein biosynthesis in cottonseed is governed by distinct regulatory mechanisms. This independence opens opportunities for improving protein content as a standalone trait, without compromising fiber or oil quality. The consistency of protein accumulation under stress, as seen in CIM 432 and others, aligns with previous findings that protein synthesis is less sensitive to water deficit than oil formation (Zafar et al., 2023). Moving forward, a deeper investigation into the genetic and molecular mechanisms controlling these divergent responses, particularly the fiber-oil trade-off—will be critical. Genomic and metabolomic analyses could reveal key pathways involved in stress adaptation and, seed, composition, aiding marker-assisted selection. Ultimately, identifying genotypes that can sustain desirable seed traits, ginning efficiency, and fiber quality under variable water, availability will contribute to more resilient and sustainable, cotton production systems in the face of water deficiency environment.

### Trait interactions and water deficiency induced trade-offs

The correlation and ANOVA analyses reveal key relationships between ginning efficiency and seed traits in cotton across irrigated and water deficiency environments, offering important implications for breeding and agronomic strategies. A consistently strong positive correlation between GR and GE under both water deficiency (r = 0.726, p< 0.05) and irrigated (r = 0.541) highlights that ginning performance is tightly coupled with energy input. This finding aligns with previous work suggesting that efficient energy transfer supports optimal fiber separation (Byler, 2006). Moreover, the moderate-to-strong positive association between GE and LP under water deficiency (r = 0.696, p< 0.05) indicates that high ginning efficiency may help sustain lint yield even under stress, reinforcing the importance of processing efficiency in water deficiency-prone regions or could be the same conditions that cause higher LP cause higher GE. Conversely, the significant negative correlation between lint fiber and oil content under both conditions (r = –0.826 and –0.841 for water deficiency and irrigated, respectively; p< 0.01) underscores a consistent physiological trade-off between structural and storage components. This fiber–oil trade-off likely reflects competition for carbon allocation during seed development and presents a core challenge for breeders aiming to simultaneously improve both fiber and seed oil contents.

Further support for these complex trait relationships demonstrates strong genotypic and environmental influences on trait expression. While most traits exhibited significant genotypic variation, GR and fiber percentage remained relatively stable across genotypes, suggesting they are less influenced by genetic plasticity. Treatment effects were significant for most traits, except GR, fuzz percentage, and fiber percentage, indicating water deficiency exerts a profound impact on seed composition and ginning performance. Notably, the significant genotype-by-treatment interaction for seed index (SI) reveals its heightened sensitivity to environmental conditions and points to its potential as a selection trait for water deficiency adaptability (Khan et al., 2018). The negative correlation between GE and SI under irrigated (r = –0.715, p< 0.05) also suggests that higher energy input may be associated with reduced seed weight, potentially due to structural changes in the seed coat under irrigated condition. Interestingly, oil content showed a moderately negative, though non-significant, correlation with GR and LP under water deficiency, hinting at metabolic shifts that prioritize fiber over lipid storage under stress. In contrast, protein content displays minimal correlation with other traits, suggesting it is regulated independently of seed fiber and oil biosynthesis. This decoupling of protein accumulation from environmental variation presents opportunities for targeted improvement. Collectively, these findings emphasize the importance of integrative breeding approaches that balance lint yield, seed quality, and processing efficiency while accounting for environmental variability, with fiber–oil dynamics emerging as critical targets for improving water deficiency tolerance in cotton.

### Implications for breeding and future directions

Our findings emphasize the critical need to select cotton genotypes that demonstrate stability in both agronomic performance and fiber quality under irrigated and non-irrigated. Genotypes such as CIM 432 emerge as a particularly strong candidate due to its consistent performance in boll retention, ginning energy efficiency, and fiber quality under water deficiency stress. In contrast, genotypes like MD 52ne and Reba P288, which exhibited greater sensitivity to water deficit, may benefit from targeted improvement strategies. Encouragingly, the general stability observed across many genotypes suggests that selecting high-quality traits under optimal conditions can confer tolerance under water deficiency, thereby streamlining breeding efforts. The independence of protein content from other traits offers an added advantage, presenting opportunities to enhance seed nutritional value without negatively affecting seed fiber or oil yields. Moreover, the lack of significant genotype-by-treatment interactions indicates that many genotypes maintain trait expression across environments, simplifying the selection process for breeders aiming to improve water deficiency tolerance. Future research should prioritize uncovering the physiological and genetic bases of these stable responses, particularly in high-performing genotypes like CIM 432. Integrating genomic, transcriptomic, and metabolomic data could accelerate marker-assisted selection (MAS) and genome-wide association studies (GWAS), enabling precise identification of loci associated with water deficiency tolerance and lint fiber-oil trade-offs. Additionally, a deeper understanding of how water deficiency conditions influence lint fiber structure and ginning energy requirements could inform the development of more energy-efficient ginning technologies, advancing both the sustainability and productivity of cotton systems under increasing environment variability.

## Data Availability

The original contributions presented in the study are included in the article/**Supplementary Material**. Further inquiries can be directed to the corresponding author.

## Supplementary material

Supplementary Figure 1 Soil moisture content (mm) at soil depth of 0-10cm **(A)**, 10-20cm **(B)**, 20-30cm **(C)**, and 30-40cm **(D)** from May to August, with blue lines representing non-irrigated (water deficiency) and orange lines representing irrigated conditions. Data points are means with error bars indicating standard deviation. Statistical significance between non-irrigated and irrigated conditions is denoted by p-values above each genotype pair (p< 0.05*, p< 0.01**, p< 0.001***, p< 0.0001****).

Supplementary Figure 2 Plant height (cm) across 10 cotton genotypes under non-irrigated (gray bars) and irrigated (green bars) conditions in late July (80 DAG). Bars represent means with error bars indicating standard deviation. Statistical significance between non-irrigated and irrigated conditions is denoted by p-values above each genotype pair (p< 0.05*, p< 0.01**, p< 0.001***, p< 0.0001****).

Supplementary Figure 3 Lint % **(A)**, fuzz % **(B)** and seed index (SI) **(C)** across 10 cotton genotypes under non-irrigated (gray bars) and irrigated (green bars) conditions. Statistical significance between non-irrigated and irrigated conditions is denoted by p-values above each genotype pair (p< 0.05*, p< 0.01**).

Supplementary Table 1 Summary of the weather and precipitation data at the Stoneville Experimental Station, MS (Elevation: 127 ft; Latitude: 33.4311° N; Longitude: 90.9108° W; Station ID: USC00228445) using records from the National Centers for Environmental Information (https://www.ncei.noaa.gov/access/past-weather/), and water deficiency conditions via the U.S. Drought Monitor (https://droughtmonitor.unl.edu/Maps/) from May 1^st^, 2024 to August 31^st^, 2024. D0 (abnormally dry), D1 (moderate drought), D2 (severe drought), D3 (extreme drought). In the irrigated treatment, plots received three times irrigated with 3 inches of water applied each time on June 26, July 17 and August 12, 2024.
