# Supplementary material for: Impact of water deficiency on cotton ginning efficiency, fiber quality, and seed composition
Source: Front Plant Sci. 2026 Feb 4;17:1761774. doi: 10.3389/fpls.2026.1761774 (PMC12913115; doi:10.3389/fpls.2026.1761774)
Supplement: Supplementary Figure 1 — Soil moisture content (mm) at soil depth of 0-10cm (A), 10-20cm (B), 20-30cm (C), and 30-40cm (D) from May to August, with blue lines representing non-irrigated (water deficiency) and orange lines representing irrigated conditions. Data points are means with error bars indicating standard deviation. Statistical significance between non-irrigated and irrigated conditions is denoted by p-values above each genotype pair (p< 0.05*, p< 0.01**, p< 0.001***, p< 0.0001****). [file DataSheet1.docx]

Supplementary figure 1: Soil moisture content (mm) at soil depth of 0-10cm (A), 10-20cm (B), 20-30cm (C), and 30-40cm (D) from May to August, with blue lines representing non-irrigated (water deficiency) and orange lines representing irrigated conditions. Data points are means with error bars indicating standard deviation. Statistical significance between non-irrigated and irrigated conditions is denoted by p-values above each genotype pair (p < 0.05*, p < 0.01**, p < 0.001***, p < 0.0001****).

Supplementary figure 2: Plant height (cm) across 10 cotton genotypes under non-irrigated (gray bars) and irrigated (green bars) conditions in late July (80 DAG). Bars represent means with error bars indicating standard deviation. Statistical significance between non-irrigated and irrigated conditions is denoted by p-values above each genotype pair (p < 0.05*, p < 0.01**, p < 0.001***, p < 0.0001****).


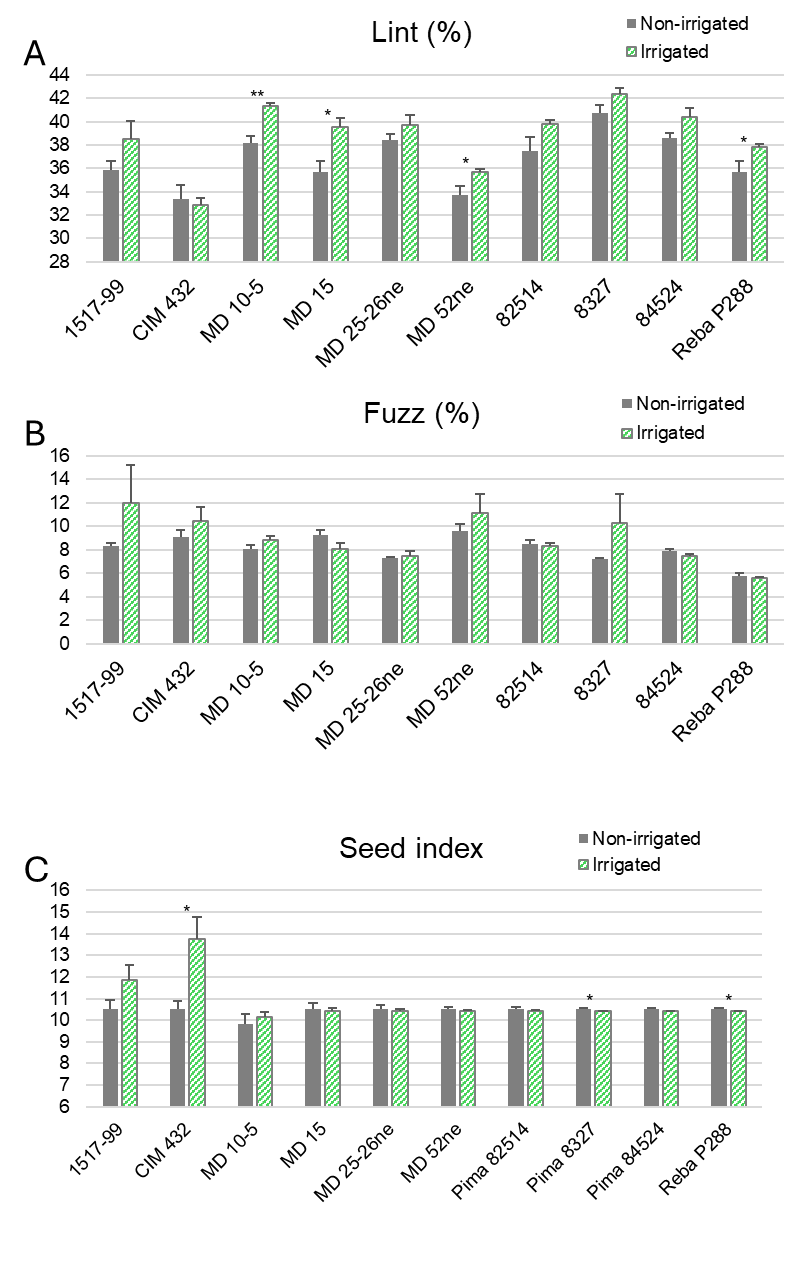


Supplementary figure 3: Lint % (A), fuzz % (B) and seed index (SI) (C) across 10 cotton genotypes under non-irrigated (gray bars) and irrigated (green bars) conditions. Statistical significance between non-irrigated and irrigated conditions is denoted by p-values above each genotype pair (p < 0.05*, p < 0.01**).

Supplementary table 1: Summary of the weather and precipitation data at the Stoneville Experimental Station, MS (Elevation: 127 ft; Latitude: 33.4311° N; Longitude: 90.9108° W; Station ID: USC00228445) using records from the National Centers for Environmental Information (<https://www.ncei.noaa.gov/access/past-weather/>), and water deficiency conditions via the U.S. Drought Monitor (<https://droughtmonitor.unl.edu/Maps/>) from May 1^st^, 2024 to August 31^st^, 2024.

D0 (abnormally dry), D1 (moderate drought), D2 (severe drought), D3 (extreme drought). In the irrigated treatment, plots received three times irrigated with 3 inches of water applied each time on June 26, July 17 and August 12, 2024.
